# Visualization of the location and level of pain in common wrist pathologies using color-coded heatmaps

**DOI:** 10.1007/s00402-022-04479-1

**Published:** 2022-06-06

**Authors:** Nicholas Moellhoff, Veronika Throner, Konstantin Frank, Ashley Benne, Sonja Adelmann, Michaela Coenen, Riccardo E. Giunta, Elisabeth Haas-Lützenberger

**Affiliations:** 1 Division of Hand, Plastic and Aesthetic Surgery, University Hospital, LMU Munich, Ziemssenstr. 5, 80336 Munich, Germany; 2 Department of Medical Information Processing, Biometry, and Epidemiology (IBE), Chair for Public Health and Health Services Research, Research Unit for Biopsychosocial Health, LMU Munich, Munich, Germany; 3 Pettenkofer School of Public Health, Munich, Germany

**Keywords:** Wrist pain, Hand surgery, Trauma surgery, CMC-1-Osteoarthritis, Ganglion, TFCC tear

## Abstract

**Background:**

Pain of the hand and wrist affects a large patient population. If the onset is unrelated to recent trauma, the first medical contact is rarely established with a specialized hand surgeon.

**Objective:**

The objective of this investigation was to (1) visualize the localization of hand pain using pain-related heatmaps in common wrist pathologies, (2) to test whether differences between these pathologies exist with regard to sociodemographic and pain-related aspects, and (3) to evaluate the major patient-reported complaints associated with the pathologies.

**Methods:**

This observational cross-sectional study included patients suffering from: thumb basal joint arthritis (CMC-1-OA), dorsal wrist ganglions, and TFCC tears. Patients marked the location of maximum pain projection on hand graphics depicting the outline of the palmar and dorsal hand. Color-graded frequency heat maps were generated for the wrist pathologies investigated. Daily life impairments were assessed and clustered into groups of functions/activities.

**Results:**

120 patients with a mean age of 44.3 years were investigated. The diagnostic groups showed significant differences regarding the level and location of pain, as well as daily life impairments. Patients with CMC-1-OA presented with increased pain levels compared to patients with dorsal wrist ganglions and TFCC tears. Daily life impairment was rated highest when household chores were adversely affected, and sport activities were symptomatic/painful. All groups showed significant skin surface pain projection, which was visualized in heatmaps. While general trends in pain localization were visible, pain levels were also reported distal/proximal and palmar/dorsal to the pathology.

**Conclusions:**

Knowledge of main demographic parameters, pain projection, and degree of impairment in daily activities can help physicians to narrow differential diagnosis of wrist pain during first patient contact. Patients should then be referred to hand surgeons for specialist examination, to further differentiate the origin of the pain.

## Introduction

Hand and wrist pain are common musculoskeletal disorders with a combined prevalence of approximately 10% in the general population [1]. Wrist pain alone is the fourth most common site of pain in the upper extremity. Numbers are considered highest in people who perform physically demanding manual labor and in those who are physically active [2]. A variety of  health-care practitioners are confronted with patients suffering from acute or chronic wrist pain. These include general practitioners, orthopedic surgeons, physiotherapists, occupational therapists, rheumatologists, trauma surgeons, as well as plastic and hand surgeons.

Management of wrist pain can be challenging and differential diagnosis often correlates with extensive work-up [3]. In addition to physical examination and diagnostic imaging, basic anamnestic evaluation can guide practitioners toward the correct diagnosis [4]. Much information can be deduced from patients’ gender, age, daily activity, as well as the self-reported level and localization of pain. Pain is primarily evaluated and monitored at rest and under stress using numeric rating or visual analogue scales (NRS/ VAS) on a range of 0 to 10 [5]. In addition, the amount and frequency of pain medication required daily provides further detail on pain severity. It is, however, especially the location of pain (volar/ dorsal, as well as ulnar/radial) which helps narrow the differential diagnosis. For example, ulnar wrist pain can point to TFCC tear, ulna impaction syndrome, or lunotriquetral ligament tear [6], while radial wrist pain can point to carpometacarpal osteoarthritis (CMC-1-OA) or De Quervain’s tenosynovitis [7] and dorsal pain to a ganglion cyst or scapholunate ligament tear [8].

This study investigated an innovative approach to visualize pain associated with three frequent wrist pathologies: CMC-1-OA, dorsal wrist ganglions, and TFCC tears. The specific aims were (1) to assess the level of pain and illustrate the self-reported location of pain on the skin surface, (2) to test whether there are differences between the diagnostic groups with regard to sociodemographic and pain-related aspects, and (3) to evaluate the major complaints associated with the pathologies.

## Materials and methods

### Study design

This observational cross-sectional study was designed as a retrospective study to explore and visualize the level and location of stress-induced pain in patients with three common wrist pathologies. The study was conducted at a level 1 hospital in Germany (Division of Hand, Plastic and Aesthetic Surgery, University Hospital, LMU Munich).

### Sample

Patients who presented with any of the three following common hand surgical diagnoses were included in this study: thumb basal joint arthritis (CMC-1-OA), dorsal wrist ganglions, and TFCC tears. Exclusion criteria included a recent operation in the specific area. Data were collected anonymously. The study was conducted between January 2020 and April 2021 and in accordance with the principles of the Declaration of Helsinki 1996, regional laws and good clinical practice. Prior to initiation, ethical approval was waived by the Institutional Review Board of the medical faculty LMU Munich (Ref.-Nr.: 22-0104).

### Assessments and outcomes

**Fig. 1:**
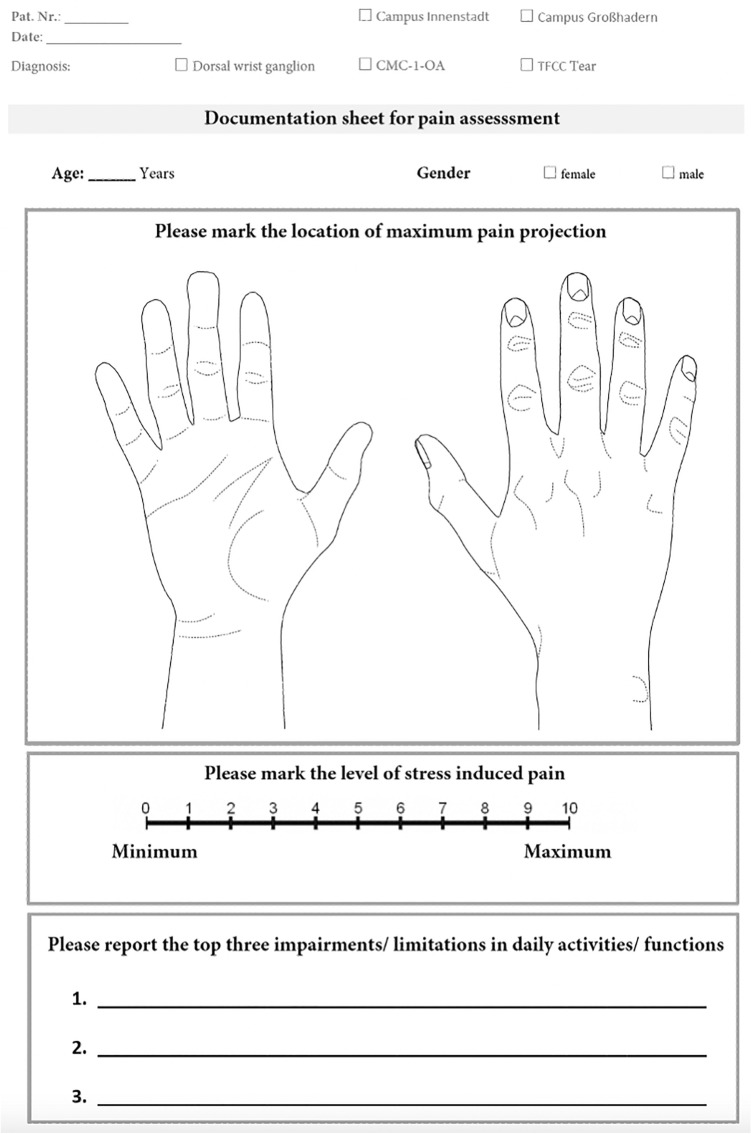
Documentation sheet (translated to English) utilized for the assessment of pain levels and location of pain

**Fig. 2:**
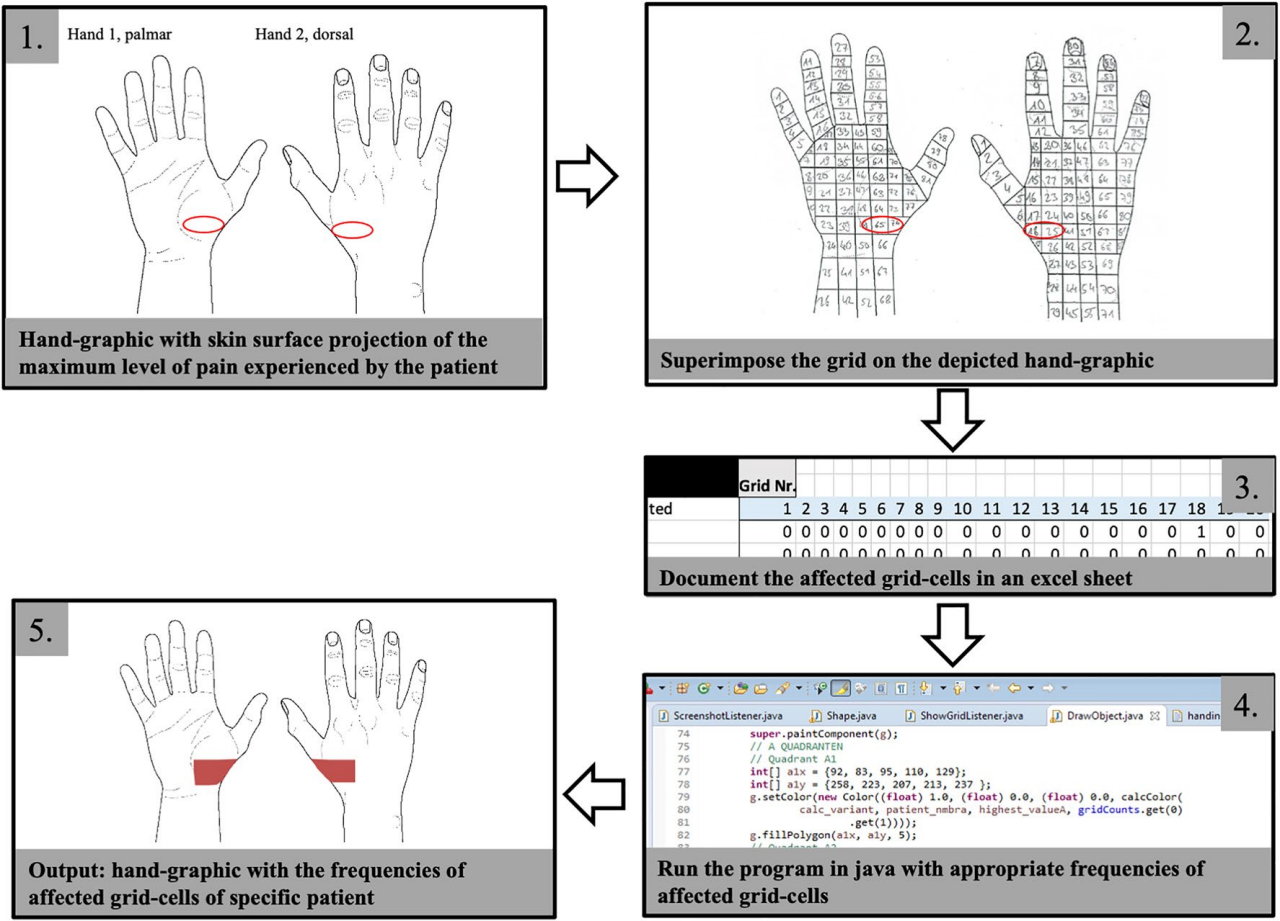
Exemplary workflow on hand graphics to visualize frequencies of affected areas on one specific patient. Patients mark the location of maximum pain projection on hand graphics. A grid with numbered cells is adapted to the hand-graphic, superimposing the depicted skin surface projection of pain. The cells containing the patient’s mark are recorded in an excel sheet. Images are created by processing and visualizing affected cells using a program written in java 1.7

Data were collected using a specifically designed documentation sheet (Fig. 1), routinely used for patients presenting to the outpatient clinic. In addition to demographic variables such as age, gender, and diagnosis, patients reported the top three impairments/limitations in daily activities/functions by answering free text questions. Stress-induced pain was measured on a 11-point Numerical Rating Scale (NRS). Heatmaps depicting the skin surface projection of pain were generated as follows: First, patients marked the location of maximum pain projection on hand graphics depicting the outline of the palmar and/or dorsal hand (see Figs. 1, 2, picture 1). Next, a grid with unique numbered cells was adapted to the hand-graphic, superimposing the depicted skin surface projection of pain (see Fig. 2, picture 2). Then, the cells containing the patient´s mark were recorded in an excel sheet (see Fig. 2, picture 3). Images were created by processing and visualizing affected cells using a program written in java 1.7 (http://www.oracle.com/) (see Fig. 2, picture 4). The major components of the program utilized methods and classes of the two graphical programming libraries Abstract Window Toolkit (AWT) and Swing.

For each of the hand illustrations (palmar and dorsal), equal grid cells of all included patients were summed up according to their diagnosis. Then, their frequencies were encoded by the gradation of red color intensity. The darker the color, the more patients reported pain in this area of the hand (Fig. 5).

### Statistical analysis

Data are presented as means with their respective standard deviation (1 SD), or as absolute and relative frequencies. Data were tested for normal distribution using the Shapiro–Wilk normality test and through visual inspection of Q–Q plots. Differences between diagnostic groups on a continuous dependent variable were calculated using one-way analysis of variance (ANOVA). Homogeneity of variances was calculated using Levene’s test of homogeneity of variances. Differences between diagnostic groups on dichotomous or multinomial dependent variables were assessed using the Chi-square or Fisher’s exact test as deemed appropriate. Answers to free text questions were clustered into groups of activities/functions and reported as absolute and relative frequencies. The level of significance was set at a probability level of *p* ≤ 0.05. All calculations were performed using SPSS Statistics 29 (IBM, Armonk, NY, USA).

### Results

In total, 120 patients (33 males; 83 females; 4 missing) with a mean age of 44.3 ± 15.8 years participated in this study. Forty-three (36%) patients presented with a TFCC tear, 42 (35%) with CMC-1-OA, and 35 (29%) with a dorsal wrist ganglion. Overall, the mean level of pain across the entire patient population was 3.6 ± 2.4 (out of 10). Eighteen (15%) patients suffered from palmar, 72 (60%) from dorsal and 30 (25%) from both palmar and dorsal pain.

### Gender

**Fig. 3:**
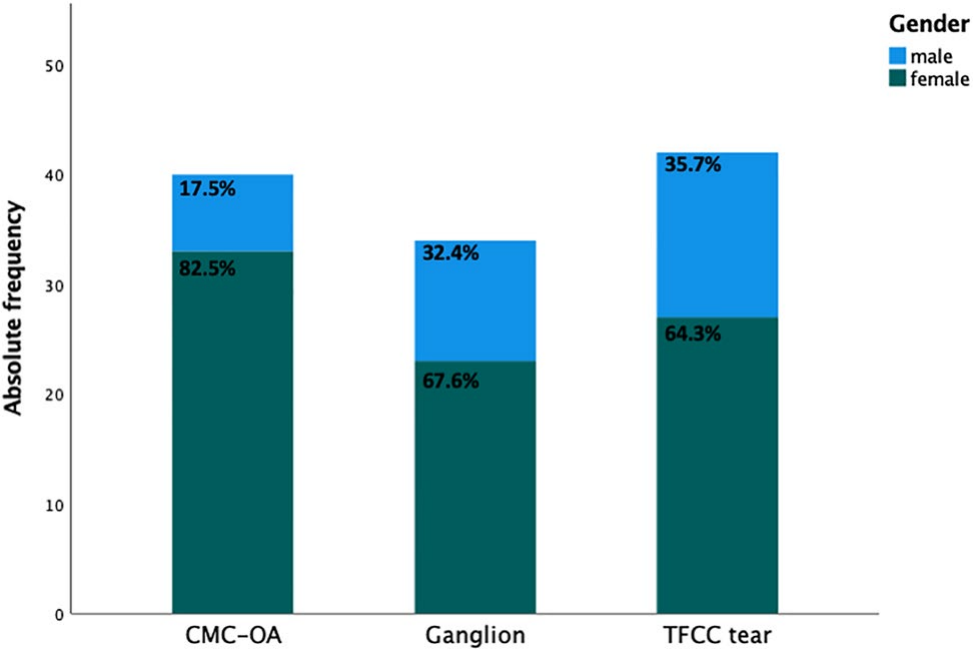
Bar graph depicting gender distribution according to the diagnostic groups. Data depicted as absolute and relative frequencies

Gender distribution was homogenous between groups (*p* = 0.157). All groups included pre-dominantly females, with numbers being highest among the CMC-1-OA group (*n* = 33, 83%) (Fig. 3).

### Age

Age differed significantly between the diagnostic groups (*p* < 0.001). Those suffering from CMC-1-OA were eldest with a mean age of 58.7 ± 6.8 years, followed by patients suffering from a TFCC tear with a mean age of 40.4 ± 14.2 years and a dorsal wrist ganglion with a mean age of 32.4 ± 12.5 years, respectively (Table 1).

**Table 1:** Distribution of patients’ mean age across the different diagnostic groups. Data depicted as mean (SD)

	CMC-arthritis	Ganglion	TFCC	Total	*P* value
Mean age	58.7	32.4	40.4	44.3	< 0.001
SD	6.8	12.5	14.2	15.8	

*SD* standard deviation

### Level of pain

**Fig. 4:**
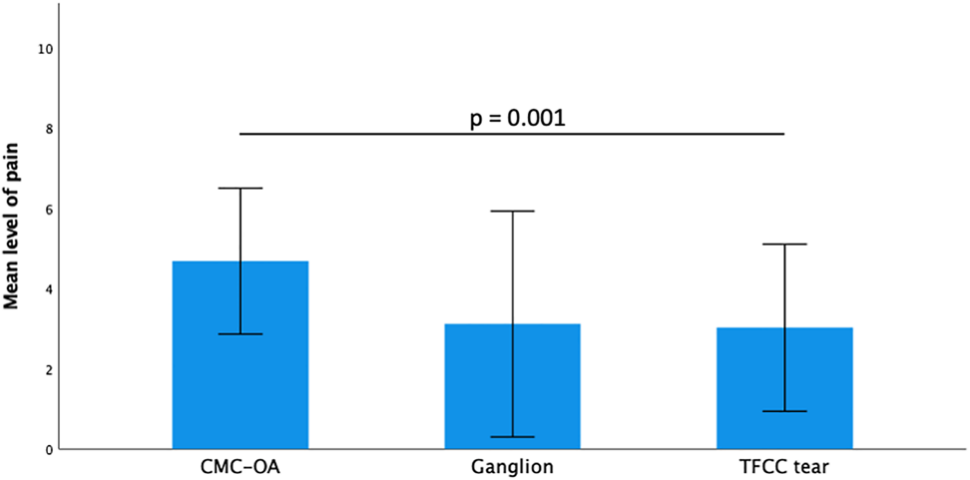
Bar graph depicting the distribution of patients’ mean level of stress-induced pain as assessed on a 11-point NRS across the different diagnostic groups. Data depicted as mean (SD)

Pain levels showed significant differences between the three diagnostic groups (*p* = 0.001). The mean level of stress-induced pain was highest in patients with CMC-1-Osteoarthritis (4.7 ± 1.8), followed by patients with a dorsal wrist ganglion (3.11 ± 2.8) and patients with a tear of the TFCC (3.02 ± 2.1) (Fig. 4).

### Location of pain

While in all diagnostic groups. The most frequent location of pain was the dorsum of the hand, there were significant differences in regards to location overall (*p* = 0.019). Patients with CMC-1-Osteoarthritis suffered most frequently from pain in the dorsal hand (*n* = 18, 43%), followed by combined palmar and dorsal pain (*n* = 14, 33%) and lastly palmar pain only (*n* = 10, 24%). In comparison, patients with a dorsal wrist ganglion reported pain in the dorsum of the hand in 80% (*n* = 28), both palmar and dorsally in 11% (*n* = 4) and isolated palmar pain in only 9% (*n* = 3) of cases. Patients suffering from a TFCC tear had pain in the dorsum of the hand in 61% (*n* = 26) of cases, both palmar and dorsal pain in 28% (*n* = 12), and palmar pain only in 12% (*n* = 5) of cases (Table 2).

**Table 2:** Overall localization of pain with regard to the three diagnostic groups

	CMC-arthritis		Ganglion		TFCC		Total		*P* value
	*n*	%	*n*	%	*n*	%	*n*	%	
Palmar	10	23.8	3	8.6	5	11.6	18	15.0	0.019
Dorsal	18	42.9	28	80.0	26	60.5	72	60.0	
Both	14	33.3	4	11.4	12	27.9	30	25.0	
Total	42	100.0	35	100.0	43	100.0	120	100.0	

Data depicted as absolute and relative frequencies. Percentages are calculated based on the total number of values within the diagnostic groups

**Fig. 5:**
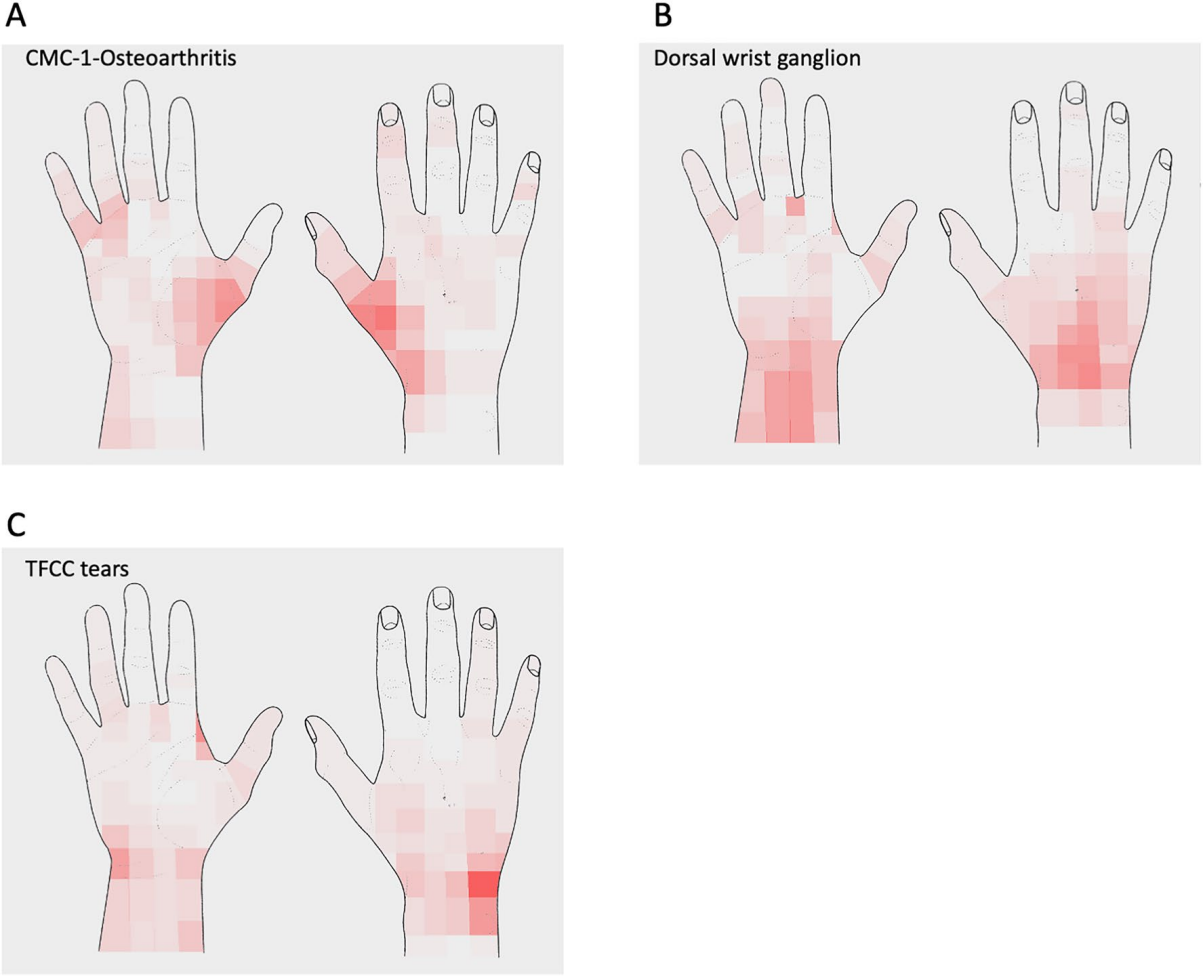
Hand graphics (**A** CMC-1-Osteoarthritis; **B** Dorsal wrist ganglion; **C** TFCC tear) with color-coded frequencies of pain projection at the skin surface

The skin surface landmarks of pain location and the color-grading with respect to the frequency reported are highlighted in Fig. 5 for the respective groups.

### Activity

**Fig. 6:**
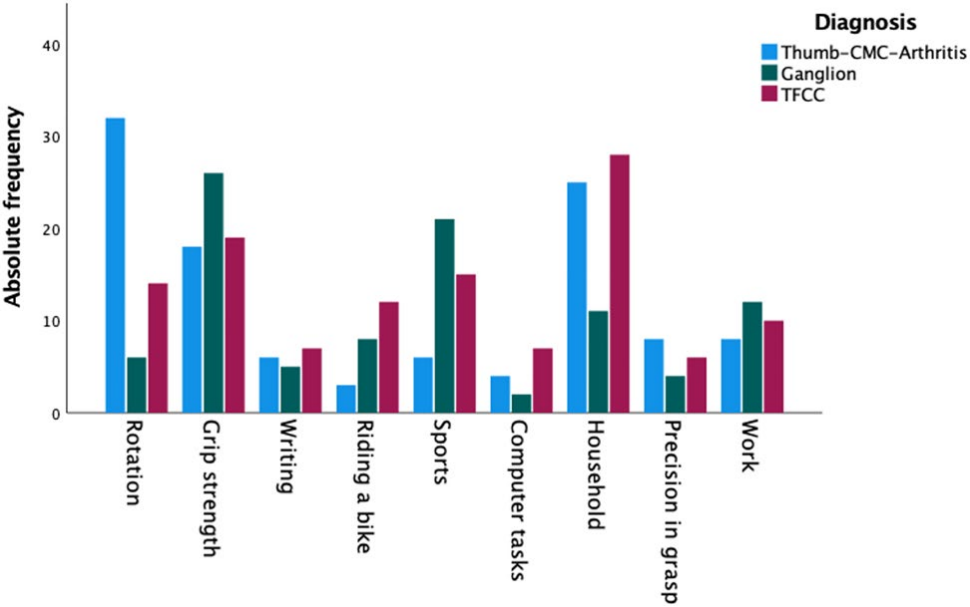
Bar graph depicting clustered results of impairments/limitations in daily activities/functions reported by patients according to the diagnostic groups. Data depicted as absolute frequencies

Overall, patients reported limitations in daily activities due to wrist pathologies most frequently during daily household activities (20%), when performing sports (13%), at work (9%), when riding a bike (7%), during writing (6%), and computer tasks (4%), as well as functional impairments in activities requiring grip strength (20%), rotation (16%), or precision in grasp (6%). Detailed information is provided in Table 3, Fig. 6. Patients with CMC-1-Osteoarthritis reported functional impairment during rotation (*n* = 32, 29%) most frequently, followed by limitations in household activities (*n* = 25, 23%). The two most common impairments for patients with dorsal wrist ganglion were performing tasks requiring strength of grip (*n* = 26, 27%), as well as sports (*n* = 21, 22%). Patients with TFCC tears complained of limitations during daily household activities (*n* = 28, 24%), followed second by tasks requiring strength of grip (*n* = 19, 16%).

**Table 3:** Overview of clustered responses of patients on impairments/ limitations in daily activities/functions according to the diagnostic groups

		CMC-Arthritis	Ganglion	TFCC	Total
Rotation	*n*	32	6	14	52
	%	29.1%	6.3%	11.9%	16.1%
Grip strength	*n*	18	26	19	63
	%	16.4%	27.4%	16.1%	19.5%
Writing	*n*	6	5	7	18
	%	5.5%	5.3%	5.9%	5.6%
Riding a bike	*n*	3	8	12	23
	%	2.7%	8.4%	10.2%	7.1%
Sports	*n*	6	21	15	42
	%	5.5%	22.1%	12.7%	13.0%
Computer tasks	*n*	4	2	7	13
	%	3.6%	2.1%	5.9%	4.0%
Household	*n*	25	11	28	64
	%	22.7%	11.6%	23.7%	19.8%
Precision in grasp	*n*	8	4	6	18
	%	7.3%	4.2%	5.1%	5.6%
Work	*n*	8	12	10	30
	%	7.3%	12.6%	8.5%	9.3%
Total	*n*	110	95	118	323
	%	100.0%	100.0%	100.0%	100.0%

Rotation including opening a jar/ bottle, turning keys; Grip strength including lifting/holding/grasping or pushing heavy objects; Household including cooking, cleaning, gardening; precision in grasp including opening/closing of buttons/zippers

Percentages are calculated based on the total number of values within the diagnostic groups

### Discussion

This study investigated demographic variables, level, and location of pain as well as impairments and limitations in daily activities and functions in patients suffering from three common wrist pathologies. Using an unprecedented data collection manner, skin surface landmarks of pain location in patients suffering from CMC-1-Osteoarthritis, dorsal wrist ganglion, and TFCC tear were illustrated using color-coded heatmaps. While a plethora of technical devices, i.e., magnetic resonance imaging (MRI) or computer tomography (CT) scans are utilized to determine the etiology of wrist pain, medical history and clinical examination remain of utmost importance in diagnostics. This remains to be the case, especially pertaining to increasing economic pressure from health insurance providers and hospitals alike. A thorough specialist clinical examination can reduce unnecessary radiation, reduce financial strain, and allow efficient time management of both patients and surgeons. The results of this study help to narrow the potential diagnosis of patients with wrist pain and can guide practitioners toward initiating the correct diagnostic examinations. However, the results also highlight that there remains a level of ambiguity in self-reported location of pain, highlighting the need for specialist examination and diagnostic work-up by specialized hand surgeons.

Patients with CMC-1-Osteoarthritis were as expected eldest, included mostly females, and reported the highest level of pain out of all investigated subjects. Naturally, osteoarthritis is an age-related disease [9], which is reflected by the study population with a mean age of 58.7 ± 6.8 years. The predominance in females can be correlated to articular age- and gender-related changes of the CMC-1-joint, contributing to severer joint space narrowing in women due to hormonal changes and as a result of menopause [10–12]. Our study results show that patients suffering from CMC-1-OA have difficulties in localizing pain in one specific area of the hand. While pain was reported pre-dominantly at the radial dorsal hand, pain was also outlined in the radial palmar hand or both. The heterogenic pain projection reported by the patients matches the CMC-1-joint’s capsule innervation either via the radial, ulnar, or median nerve [13]. The ambiguity in pain location reported by patients may, however, complicate formulating a correct diagnosis. Hence, referral to a specialized hand surgeon is warranted. The most common differential diagnosis to consider in patients reporting the aforementioned pain projection is De Quervain´s tenosynovitis. Differentiation between these diagnoses is possible using medical history and clinical examination, exploring the patients age, occupation, daily activities, and by performing the pathognomonic Finkelstein’s test [14].

Patients suffering from a dorsal wrist ganglion, which originates in most cases from the scapholunate interosseous ligament [15], reported pain in the dorsal wrist in a majority of cases. This location can again be explained by the sensory innervation of the dorsal wrist capsule. The dorsal wrist capsule is innervated in most instances by the posterior interosseous nerve (PIN) of the radial nerve [16]. Even in patients without a palpable or visible protuberance of the ganglion, which simplifies diagnosis, our investigations help to narrow the possible differentials. Most typically, patients with dorsal wrist ganglions are young females with chief complaint being pain while performing activities requiring grip strength and sports (i.e., body weight exercises). The pain level to be expected is relatively low with an average of 3.11 ± 2.8, as measured on the NRS. Especially, if the ganglion is occult and thus cannot be seen or palpated, it is important to obtain a complete medical history and thorough clinical examination. Knowledge of patients’ age, pain location, and restricted daily activity can then help to identify the correct diagnosis. In addition to this, ultrasound imaging can guide toward identifying a ganglion, without the need for X-ray radiography [17, 18]. Hand surgeons are trained in ultrasound investigation of the hand- and wrist. Patients presenting with aforementioned clinical history should be seen by a specialized hand surgeon for further clinical work-up.

Regarding TFCC tears, medical history helps to identify whether the patient suffers from an acute traumatic one-sided tear which is pre-dominantly found in young male patients, or a degenerative bilateral cause due to ulna variance, pre-dominantly found in female patients. The pain level to be expected is lower than that of CMC-1-OA or dorsal ganglion cyst. Bilateral conventional X-ray imaging is required in degenerative cases with ulna variance, while acute traumatic injuries require MR-arthrography or at minimum a conventional MRI [19–21].

While, overall, the findings of this investigation are not novel per se, this study provides quantification and visualization of patient-reported pain levels and locations for the first time and using a novel approach. The data demonstrate that the information on age, gender, pain level, and location as well as impaired daily activities can guide the clinician to narrow possible differential diagnosis to the most common wrist pathologies. Color-coded heatmaps can provide guidance especially for young hand surgeons, orthopedic surgeons not specialized in hand surgery, as well as general practitioners in differential diagnosis of wrist pain. However, due to the remaining ambiguity in reported pain location, referral to a specialized hand surgeon is recommended. A multitude of diagnostic measures exist to further narrow the diagnosis of acute or chronic wrist pain. Especially radiological imaging is regarded paramount in determining the underlying disease. However, there are several radiographic views available (including stress views, dynamic investigations, and imaging using contrast agents) to best describe the suspected diagnosis. Hand surgeons are trained in physical examination of the hand and have knowledge of the radiologic assessments required. In terms of cost-effectiveness, after the initial narrowing of diagnosis by clinical investigation, patients should be referred to hand surgeons who can then decide upon further diagnostic measures for further clarification of the underlying disease.

This study is not free of limitations. Distribution of standardized questionnaires, i.e., DASH, MHQ, and EQ-5D, and the evaluation of objectifiable measurement parameters such as grip strength or range-of-motion would have allowed further differentiation with regard to loss of function and the impact of the pathologies on quality of life.

## Conclusions

Color-coded heatmaps provide an innovative method to visualize the skin surface pain projection of common wrist pathologies. Knowledge of patients’ age, gender, level, and location of pain, as well as degree of restriction in daily activity allows physicians to narrow differential diagnosis. However, there remains ambiguity in self-reported surface projection of pain, thus potentially obscuring the correct diagnosis. Hence, after the initial clinical investigation and narrowing of possible diagnoses, patients with wrist pain should be referred to specialized hand surgeons who can then decide upon further diagnostic measures.
